# *Salmonella enteridis* Septic Arthritis: A Report of Two Cases

**DOI:** 10.1155/2013/642805

**Published:** 2013-10-22

**Authors:** Esat Uygur, Krishna Reddy, Feyza Ünlü Özkan, Salih Söylemez, Özlem Aydin, Serkan Şenol

**Affiliations:** ^1^Department of Orthopedics and Traumatology, Istanbul Medeniyet University, Göztepe Training and Research Hospital, Istanbul, Turkey; ^2^Royal Orthopaedic Hospital, Birmigham, UK; ^3^Department of Physical Therapy and Rehabilitation, Fatih Sultan Mehmet Training and Research Hospital, Istanbul, Turkey; ^4^Department of Infectious Diseases, Istanbul Medeniyet University, Göztepe Training and Research Hospital, Istanbul, Turkey; ^5^Department of Pathology, Istanbul Medeniyet University, Göztepe Training and Research Hospital, Istanbul, Turkey

## Abstract

*Introduction*. Nontyphoidal salmonellosis causes significant morbidity, is transmitted via fecal-oral route, and is a worldwide cause of gastroenteritis, bacteremia, and local infections. Salmonella is a less common etiologic factor for septic arthritis compared with other gram-negative bacteria. *Cases*. We present two septic arthritis cases with *Salmonella enteridis* as a confirmed pathogen and also discuss the predisposing factors and treatment. *Discussion*. Septic arthritis is an orthopedic emergency. The gold standard treatment of septic arthritis is joint debridement, antibiotic therapy according to the culture results, and physiotherapy, which should start in the early postoperative period to prevent limitation of motion. *Salmonella* is an atypical agent for septic arthritis. It must be particularly kept in mind as an etiologic factor for the acute arthritis of a patient with sickle cell anemia and systemic lupus erythematosus. Clinicians should be cautious that the white blood cell count in synovial fluid can be under 50.000/mm^3^ in immune compromised individuals with septic arthritis. The inflammatory response can be deficient, or the microorganism may be atypical. *Conclusion*. Atypical bacteria such as Salmonella species in immune compromised patients can cause joint infections. Therefore, Salmonella species must always be kept in mind for the differential diagnosis of septic arthritis in a clinically relevant setting.

## 1. Introduction

Nontyphoidal salmonellosis causes significant morbidity, is transmitted via fecal-oral route, and is a worldwide cause of gastroenteritis, bacteremia, and local infections [1]. When compared with other gram-negative bacteria, *Salmonella* is a less common etiologic factor for septic arthritis. However, underlying conditions such as malignancy, hemoglobinopathy, diabetes mellitus, human immunodeficiency virus infection, history of intraarticular trauma, surgical operations, and other immune suppressive states predispose an individual to salmonella arthritis [2, 3]. We present two *Salmonella* septic arthritis cases and also the predisposing factors and treatment.

### 1.1. Case 1

A 38-year-old male patient was admitted via the emergency department with knee pain of 15 days duration and new onset fever. His history revealed that a general practitioner assessed him when his pain first started. There was no history of preceding trauma. He was treated symptomatically. Painful joint stiffness and effusion were noted on physical examination. However, the patient was afebrile (36.8°C), and no local warmth was detected around the knee joint either. He was observed to have many tattoos on his body. His previous medical history also included the use of prednisolone (24 mg/day) and azathioprine (2 × 50 mg/day) for the treatment of pemphigus vulgaris and systemic lupus erythematosus. His social history included smoking tobacco cigarettes and also using alcohol. Cloudy synovial fluid was obtained with knee joint aspiration. Automatic cell count and microscopic examination results were as follows: white blood cell (WBC) count: 83.100/mm^3^ (90% PMN) and dense leukocytes in each high-power field. Laboratory studies also yielded the following results: C-reactive protein (CRP): 9,9 mg/dl (0-1 mg/dl), WBC: 12.000/mm^3^ (4.000–10.000/mm^3^), and erythrocyte sedimentation rate (ESR): 82 mm/h (<20 mm/h).

All of these findings were considered as consistent with septic arthritis of the knee. Open debridement was performed. The drain could be removed at postoperative sixteen day due to prolonged drainage. Culture and antibiotic susceptibility testing of the intraoperative knee joint fluid sample results were obtained on postoperative seventh day and demonstrated *Salmonella enteritidis*, which was sensitive to ciprofloxacin. The patient received intravenous ciprofloxacin for the first week. Antibiotic therapy was continued with oral ciprofloxacin for three more weeks. Range of motion and isometric quadriceps exercises were started on the first postoperative day and continued for six weeks. At the last followup visit at twelve months postoperatively, the patient displayed full range of motion without any pain in his knee joint.

### 1.2. Case 2

A 54-year-old male patient with a known history of diabetes mellitus and chronic lymphocytic leukemia was admitted to the emergency department with fever, tremor, cold sensation, productive cough, and fatigue. At presentation, his laboratory results were as follows: WBC: 135,000/mm^3^ (4.000–10.000/mm^3^), CRP: 2.6 mg/dl (0-1 mg/dl), and ESR: 98 mm/h (<20 mm/h). He was admitted to the hospital with the diagnosis of pneumonia. He was started on moxifloxacin on the recommendation of the infectious disease department. On the second day of his hospitalization, he was referred to the orthopedics and traumatology department due to new onset right hip pain and not being able to bear weight on his right side. Examination revealed restricted and painful flexion and internal rotation in his right hip joint. X-ray examination of the right hip was unremarkable except for slight degenerative changes consistent with his age. However, ultrasonography of the hip demonstrated increased intra-articular fluid in the hip joint. Purulent fluid was aspirated from hip joint under ultrasonographic guidance. Leukocyte count of the joint fluid was 3.840/mm^3^ with 90% polymorphonuclear leucocytes. He underwent arthrotomy and debridement of the right hip immediately with the diagnosis of septic arthritis. Culture and antibiotic susceptibility testing of the hip joint fluid sample results revealed *Salmonella enteritidis*, which was sensitive to ciprofloxacin, on the postoperative seventh day. He was treated with intravenous ciprofloxacin administration for the first three weeks after the culture results, and orally for three further weeks it was performed. Physiotherapy was initiated at the fourth postoperative day after removing the drainage catheter. In the last follow-up visit at fourteen months postoperatively, he was free of pain, and there was no restriction of motion in his right hip joint.

## 2. Discussion

Septic arthritis is an orthopedic emergency. The gold standard of treatment is joint debridement and antibiotic therapy according to the culture results. Physiotherapy should be initiated early in the postoperative period to prevent limitation of motion. Smith et al. reported that enzymatic destruction begins by the eighth hour after the inoculation. By the 48th hour, 40% of the glycosaminoglycan is lost, and collagen breakdown occurs in a period of few days in septic arthritis [4]. Several studies have shown that the duration between the beginning of symptoms and surgical intervention is the most important prognostic factor for septic arthritis [5, 6]. Wirtz et al. reported better functional results for cases treated with surgery before the fifth day of symptoms [5]. The drainage of the joint prevents not only the adverse effects of high pressure on cartilage nutrition but also provides the removal of necrotic materials, bacterial debris, and enzymatic products [7]. Debridement also prevents intracapsular fibrosis, while early physical therapy is essential to maintain the range of motion [8]. Therefore, we suggest physical therapy on the first postoperative day with isometric quadriceps exercises to prevent muscle atrophy and active assisted range of motion exercises.

*Salmonella* is an atypical agent for septic arthritis. It must particularly be borne in mind as an etiologic factor for acute arthritis patient with sickle cell anemia and systemic lupus erythromatosus [9]. Gerona and Navarra reviewed twelve systemic lupus erythromatosus patients who had salmonella infection. Five of these patients developed septic arthritis, and only one of them was a culture positive for *Salmonella enteritidis* [10].

WBC counts in the synovial fluid are typically above 50.000/mm^3^ in septic arthritis. McCuthan and Fisher demonstrated that 50% of patients with positive culture results may have 28.000/mm^3^ or less WBC count in the synovial fluid. However, these patients were immune compromised [11]. In our second case, WBC count in the synovial fluid was 3.840/mm^3^ with 90% polymorphonuclear leucocytes. Raised WBC count (135.000/mm^3^) at presentation was associated with coexisting chronic lymphocytic leukemia. Taking into consideration the patient's history, immune suppression, and the purulent character of the joint fluid, septic arthritis was suspected.

Clinicians should be cautious that the WBC count in the synovial fluid can be below 50.000/mm^3^ in immune compromised individuals with septic arthritis. The inflammatory response can be deficient or the microorganism can be atypical. Virulence of salmonella is different from staphylococcus species since local or systemic fever is usually not observed. Restriction of joint motion, which is classically seen in the early period of septic arthritis, can be delayed in salmonella species. There is a subtle or no increase in warmth around superficial joints such as the knee. These cases require a high index of suspicion in the interest of time and in initiating appropriate treatment.

*Salmonella enteritidis* is mostly sensitive to fluoroquinolones and third generation cephalosporins. On the other hand, it is resistant to first generation cephalosporins that we generally prefer as an empirical treatment at the early postoperative period while waiting for culture results.

We emphasize that joint infections can be caused by atypical bacteria like salmonella species in immune compromised individuals. These patients may present atypical clinical and laboratory findings. Therefore, salmonella species must always be borne in the differential diagnosis of septic arthritis in a clinically relevant setting.
